# Supplementary material for: The causal relationship between the human gut microbiota and pyogenic arthritis: a Mendelian randomization study
Source: Front Cell Infect Microbiol. 2024 Nov 26;14:1452480. doi: 10.3389/fcimb.2024.1452480 (PMC11629706; doi:10.3389/fcimb.2024.1452480)
Supplement: Supplementary file 1 [file DataSheet1.zip › mendelian test/ebi-a-GCST90027749.csv_forest.pdf]

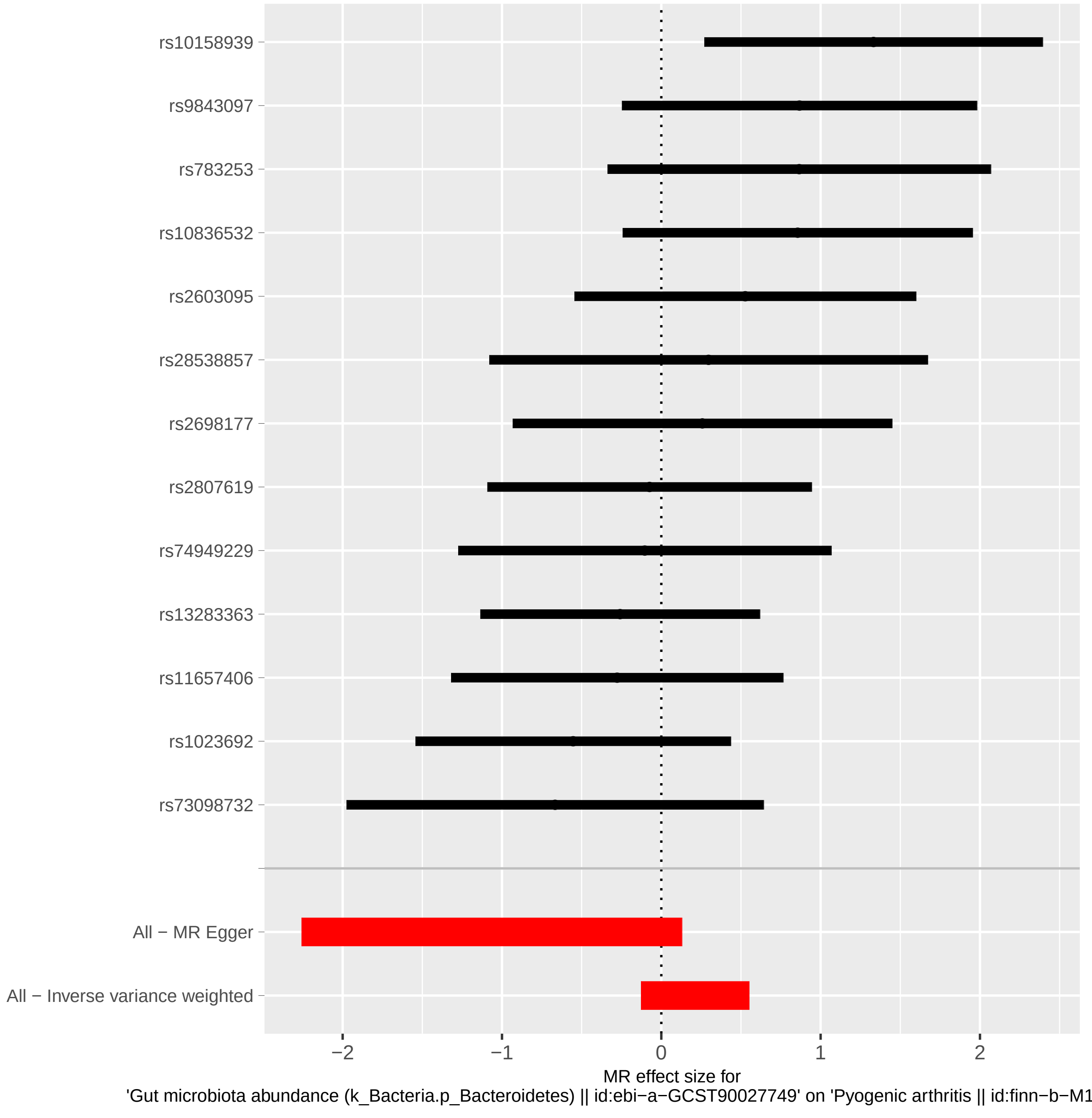
rs10158939
rs9843097
rs783253
rs10836532
rs2603095
rs28538857
rs2698177
rs2807619
rs74949229
rs13283363
rs11657406
rs1023692
rs73098732
All − MR Egger
All − Inverse variance weighted
−2
−1
0
1
2
MR effect size for
'Gut microbiota abundance (k_Bacteria.p_Bacteroidetes) || id:ebi−a−GCST90027749' on 'Pyogenic arthritis || id:finn−b−M1
